# Evaluation of depression and obesity indices based on applications of ANOVA, regression, structural equation modeling and Taguchi algorithm process

**DOI:** 10.3389/fpsyg.2023.1060963

**Published:** 2023-02-22

**Authors:** Nur Anisah Mohamed, Ayed R. A. Alanzi, Noor Azlinna Azizan, Suzana Ariff Azizan, Nadia Samsudin, Hashem Salarzadeh Jenatabadi

**Affiliations:** ^1^Faculty of Science, Institute of Mathematical Sciences, Universiti Malaya, Kuala Lumpur, Malaysia; ^2^Department of Mathematics, College of Science and Arts in Gurayat, Jouf University, Gurayat, Saudi Arabia; ^3^College of Business Administration, Prince Sultan University, Riyadh, Saudi Arabia; ^4^Department of Science and Technology Studies, Faculty of Science, Universiti Malaya, Kuala Lumpur, Malaysia; ^5^Faculty of Social Sciences and Liberal Arts, UCSI University, Kuala Lumpur, Malaysia

**Keywords:** Taguchi, obesity and depression, mHealth apps, women’s obesity, experimental design

## Abstract

**Introduction:**

Depression and obesity are the main threat among women which have been considered by many research scholars in psychology studies. In their analysis for measuring and estimating obesity and depression they were involving statistical functions.

**Methods:**

Regression, Analysis of Variance (ANOVA), and in the last two decades Structural Equation Modeling are the most familiar statistical methods among research scholars. Taguchi algorism process is one the statistical methods which mostly have been applying in engineering studies. In this study we are looking at two main objectives. The first one is to introduce Taguchi algorism process and apply it in a case study in psychology area. The second objective is challenging among four statistical techniques include ANOVA, regression, SEM, and Taguchi technique in a same data. To achieve those aims we involved depression and obesity indices with other familiar indicators contain socioeconomic, screen time, sleep time, and usage fitness and nutrition mobile applications.

**Results and discussion:**

Outputs proved that Taguchi technique is able to analyze some correlations which are not achieved by applying ANOVA, regression, and SEM. Moreover, SEM has a special capability to estimate some hidden correlations which are not possible to evaluate them by using ANOVA, regression, and even Taguchi method. In the last, we found that some correlations are significant by SEM, however, in the same data with regression those correlation were not significant. This paper could be a warning for psychology research scholars to be more careful with involving statistical methods for measuring and estimating of their research variables.

## 1. Introduction

Over time, there has been a consistent rise in the number of people receiving a depression diagnosis. The patient’s ability to work, financial situation, and interpersonal relationships are all impacted by this mental disease (Muharam et al., 2018). Passive behaviors such as disinterest, guilt-ridden thoughts, low self-esteem, lack of sleep, poor appetite, perpetual sadness, or signs of weariness can all be indicators of depression (El-Gilany et al., 2018; Zeng et al., 2018). Being depressed on a day-to-day basis results in a major handicap that can cause mental and behavioral difficulties (Archana et al., 2017). It is very likely that this condition will have an effect on the patient’s physical well-being, which will ultimately lead to an elevated risk of morbidity and mortality (Elamoshy et al., 2018; Jia et al., 2018; Muharam et al., 2018). According to the World Health Organization (WHO), more than 300 million people worldwide experienced symptoms of depression in World Health Organization (2017). However, earlier research found that women, rather than men, were more likely to suffer from depression (Jin et al., 2016). It was shown that hormonal changes, such as those that occur during puberty, pregnancy, and menopause, were the most significant contributors to depression in women. Particularly after giving birth, a woman needs to have extra care and obtain the right kind of health care priorities because any unpleasant act can cause depression at this stage, which will be devastating to the entire family (Correa et al., 2017). In addition, a woman needs to obtain the right kind of health care priorities because any unpleasant act can cause depression at this stage.

On the other hand, the epidemic of overweight and obesity is quickly spreading, especially to younger age groups; as a result, it has become a serious concern for healthcare systems due to the tremendous economic and psychosocial load it places on those systems (Rippe, 2021). In 2017, it was estimated that 13% of the adult population globally was obese, and this number has consistently risen over the past few decades in both developed and developing countries (650 million people) (World Health Organization, 2017). Several types of cancer, heart disease, and diabetes type 2 are just a few of the many chronic illnesses for which obesity is a major risk factor.

On the other side, technology can help people reach their health goals. The Global Observatory for eHealth defines mobile health (mHealth) as the “medical and public health practice supported by mobile devices, such as mobile phones, patient monitoring devices, personal digital assistants (PDAs), and other wireless devices” (World Health Organization, 2011) has become a major focus in the delivery of health care in recent years because of its potential impact on a wide range of health outcomes (Boulos et al., 2014). With the use of mobile technology, psychosocial and health behavior therapies are now accessible to a wider range of patients, in their natural environments, and in real time (Heron and Smyth, 2010). Mobile applications, sometimes known as “apps,” enable remote access to health services by linking patients with health experts all over the world in a safe, confidential, and secure manner, with quick results (Qudah and Luetsch, 2019). There are now a great number of smartphone apps that may be downloaded to help manage the symptoms of anxiety and depression.

Healthcare professionals are turning to mobile health (mHealth) technologies (Yuan et al., 2015), such as nutrition and fitness apps (Bt Wan Mohamed Radzi et al., 2020; Taylor et al., 2022) to support women in managing obesity and depression from home. The nutritional apps engage women because they are convenient for monitoring daily food intake for healthier eating. Meanwhile, fitness apps help women chart their weight loss progress and BMI levels while following various exercises (Cho et al., 2015). Women also benefit from quick and easy access to health information and use mHealth apps to communicate with health professionals and peers.

There is some encouraging evidence of their usefulness, but this still has to be validated because the majority of them have not been planned or studied with the level of rigor required (Rathbone and Prescott, 2017). Moreover, inadequate scientific coverage, inaccurate weight-related information, lack of important evidence-based features, lack of involvement of health-care experts in the development process, overlooking behavior change techniques, and not undergoing rigorous scientific testing are some of the issues that plague the majority of commercial mobile apps for weight loss and management (Bardus et al., 2016).

### 1.1. Gaps in previous psychology studies

There are three gaps in previous psychology studies regarding measuring depression and obesity. Some previous studies just focused on estimating either obesity or depression. There are few studies also considered depression as an input for estimating obesity, or they involved obesity as an input for estimating depression. However, based on some former studies we found that there is a correlation between depression and obesity. The first gap of these types of studies is that lack of analysis for estimating both obesity and depression as two dependent variables in a single model. We full-fill this gap with introducing a single model with two dependent variables (depression and BMI) based on SEM technique. In terms statistical methods, ANOVA, simple correlation, regression, and SEM are the most familiar statistical methods among research scholar in psychology studies. These statistical modeling or even mathematical modeling techniques including neuro-fuzzy inference systems can determine the impact of significant independent (input) variables on dependent variable/s.

They are unable to demonstrate, however, which levels or groups of independent variables result in higher, lower, or nominal dependent variable (output) rate levels. Therefore, the question of what level or category of independent variables results in higher or lower dependent variables cannot be addressed by statistical or mathematical modeling techniques. Based on our research variables we could turn this question to, what level of usage nutrition and fitness apps, sleep time, screen time, and demographic leads women to have high level of depression or BMI? Therefore, Taguchi method can answer this question. The second gap of former in phycology studies that, based on best on knowledge there is lack of studies to answer this question. In other words, Taguchi technique, been applied in various engineering studies but there is scarce evidence for applying this pattern in psychology studies. The final gap is that, while they frequently used fitness and nutrition apps separately, it was uncommon to find apps that effectively treated both obesity and depression using a single model or pattern.

### 1.2. Objectives of the study

Based on above matters, this study is trying to achieve the following objectives:

**First objective:** to find out the impact of nutrition apps and physical apps usage on women’s obesity and depression with application of ANOVA. This is a common analysis which have been done in previous studies.

**Second objective:** to estimate the effectiveness of nutrition apps and physical apps (with other research variables) on women’s depression and obesity based on regression modeling. In this part of the study, we will have separates models for estimating BMI and depression.

**Third objective:** to estimate the effectiveness of nutrition apps and physical apps (with other research variables) on women’s depression and obesity in a single model based on SEM.

**Fourth objective:** to recognize the combination of levels of both types of nutrition and physical apps (with other research variables) leads to higher obesity and depression. For this objective of the study, we applied Taguchi algorithm process.

**Fifth objective:** To compare outputs of data analysis with different above statistical techniques.

## 2. Materials and methods

### 2.1. Taguchi method structure

In the Taguchi method, standard deviation and variation need to be measured for the expected value. The observed values were spread out from the expected value by a high standard deviation due to noise factors. The lower standard deviation indicates that the observed values are near the expected value due to noise factors. Observed and noise factor values can be controlled by the Signal-to-Noise (SN) ratio. The SN ratio effects noise factors on performance features and quantifies the variability (Salarzadeh Jenatabadi et al., 2016). The formula to calculate the signal to noise ratio (Manigandan et al., 2020) is given in Table 1.

**Table 1:** Signal-to-Noise (SN) application.

Optimisation type	Calculation of SN
Lower is better	$\mathrm{SN}=-10\log\frac{1}{n}\overset{n}{\underset{i=1}{\sum }}y_{i}^{2}$
Larger is better	$\mathrm{SN}=-10\log\frac{1}{n}\overset{n}{\underset{i=1}{\sum }}\frac{1}{y_{i}^{2}}$

Where, *n*, *y_i_*, and *i* shows the repetitions in the experiments. To be concise, *y* is the response factor (BMI and level of depression) and *n* is the number of observations.

The Taguchi method process analysis is as follows, which refers to the previous literature (Salarzadeh Jenatabadi et al., 2016):

**Step 1:** Identifying the Indicators.

**Step 2:** Calculate the weight of each indicator.

**Step 3:** Choosing an appropriate experimental design based on the Taguchi method.

**Step 4:** Identifying the Optimal Levels of the Indicators.

**Step 5:** Data Analysis.

**Step 6:** Assessing the Factors in the Columns of the Orthogonal Array.

**Step 7:** Introducing suitable patterns based on the optimal levels.

### 2.2. Participants

The sample size estimation was calculated using Krejcie and Morgan’s method (Chuan and Penyelidikan, 2006). The formula below is commonly used to establish the total sample size requirement (Krejcie and Morgan, 1970):


(1) $$s=\frac{\chi^{2}NP\left(1-P\right)}{d^{2}\left(N-1\right)}+\chi^{2}P\left(1-P\right)$$

In the above equation, s is the required sample size; $\chi^{2}$ is the table value of chi-square for one degree of freedom at the desired confidence level; *N* is the population size; *P* is the population proportion; and *d* is the degree of accuracy expressed as a proportion (0.05). This research subject focused on Malaysian women who lived in urban and populated cities, i.e., Kuala Lumpur, Selangor, Penang, and Johor. The data was distributed after the MCO *via* online questionnaires by sending the questionnaire links through WhatsApp to 878 respondents.

Note: Approaching to the participant were based on two data sets (a) University Malaya grant [title: New Framework and Statistical Approaches for Health Index Studies: Case Study in Malaysia (GPF066B-2018)] and (b) our previous studies data set (Bt Wan Mohamed Radzi et al., 2020, 2021).

### 2.3. Measurement

The research variables were classified into five sections: demographic information, lifestyle, BMI, depression level, and mHealth app frequency. The demographic details were measured in terms of four criteria as follows:

• Age: (a) 21–25 years old (value 1), (b) 26–30 years old (value 2), (c) 31–35 years old (value 3), and (d) over 35 years old (value 4).
• Education: (a) less than a high school diploma (value 1); (b) a high school diploma (value 2); (c) a diploma (value 3); (d) a bachelor’s degree (value 4); (e) a master’s or PhD degree (value 5).
• Job Experience: (a) no job experience (value 1); (b) less than 3 years (value 2); (c) 3–6 years (value 3); (d) 6–10 years (value 4); and (e) more than 10 years (value 5).
• Income: (a) less than RM 2,000 (value 1); (b) between RM 2,000 and RM 3,000 (value 2); (c) between RM 3,000 and RM 4,000 (value 3); (d) between RM 4,000 and RM 5,000 (value 4); (e) greater than RM 5,000 (value 5).

Lifestyle was measured based on Nakayama et al. (2001) and Khajeheian et al. (2018) research. The indicators included average working hours per day; physical activity per week; average screen time use per day (e.g., TV, smartphone, tablet); and average sleeping hours per night. These indicators were grouped as follows:

• Working hours: (a) none; (b) less than 7 h; (c) 7–8 h; (d) 8–9 h; (e) more than 9 h.
• Physical activity: (a) none; (b) once; (c) twice; (d) three times; (e) four times; (f) more than four times.
• Screen time: (a) less than 1 h; (b) 1–2 h; (c) 2–3 h; (d) 3–4 h; (e) more than 4 h.
• Sleeping hours: (a) less than 6 h; (b) 6–7 h; (c) 7–8 h; (d) 8–9 h; (e) more than 9 h.

Individuals’ BMI ranges were calculated using the standardized formula: (Weight in kilograms)/(Height in meters)/2 (Carlson et al., 2016). Respondents gave their weight and height, so we can group their BMI according to the following categories (Fu et al., 2018):

BMI classification: (a) underweight (18.5 kg/m^2^); (b) normal (18.5–23.9 kg/m^2^); (c) overweight (24.0–27.9 kg/m^2^); (d) obese (28.0 kg/m^2^).

The Center for Epidemiologic Studies Depression Scale (CES-D), created by Radloff (1977) was used to measure depression. Twenty items make up the CES-D questionnaire, each of which is graded on a 4-point Likert scale. Higher scores showed that the depression was more severe.

The frequency of respondents using mHealth apps was measured based on previous studies (Rasche et al., 2018). In this study, fitness and nutrition apps usage were indicated as follows:

The frequency with which mHealth apps are used: (a) Every day; (b) every couple of days; (c) weekly; (d) monthly; (e) never.

## 3. Results

### 3.1. ANOVA, regression, and SEM

Before Taguchi analysis, we applied ANOVA and regression as the main common statistical approaches. ANOVA is used to evaluate the total sum of squares, the sum of squares due to BMI, and the sum of squares due to depression level. Estimating significant factors on BMI and depression involves regression modeling. From the outputs of Table 2, it can be seen that there are some significant differences between the outputs of using nutrition and fitness apps.

**Table 2:** ANOVA outputs.

N-App	Depression level		Sum of squares	df	Mean square	*F*	Significant
		Between groups	137.037	4	34.259	1.939	0.102
		Within groups	15423.820	873	17.668		
		Total	15560.858	877			
	BMI	Between groups	169.461	4	42.365	1.737	0.140
		Within groups	21289.792	873	24.387		
		Total	21459.254	877			
F-App	Depression level		Sum of squares	df	Mean square	*F*	Significant
		Between groups	360.638	4	90.159	5.178	0.000
		Within groups	15200.220	873	17.411		
		Total	15560.858	877			
	BMI	Between groups	2612.257	4	653.064	40.250	0.000
		Within groups	18846.997	873	21.589		
		Total	21459.254	877			

**Figure 1:**
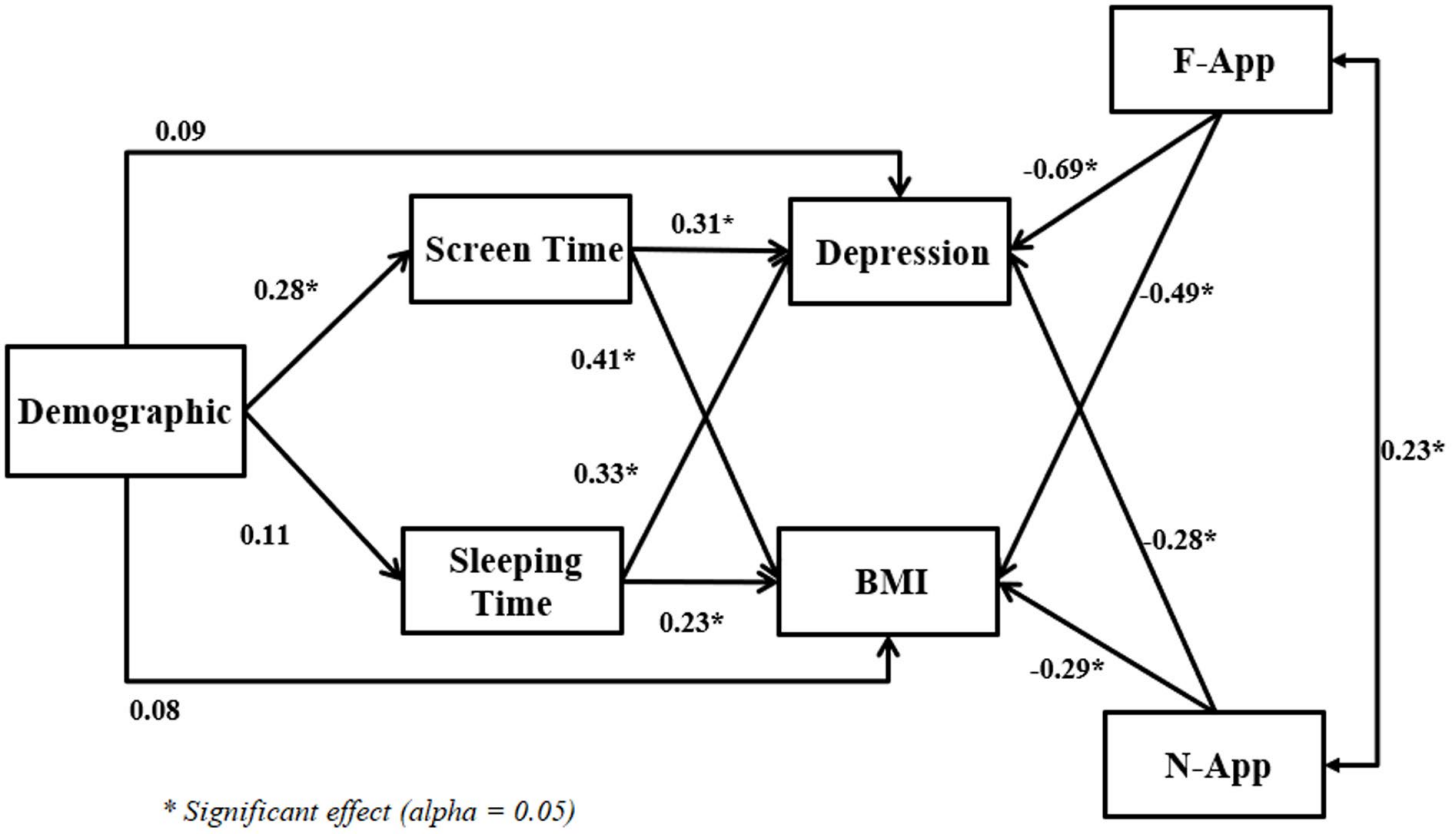
SEM outputs (dependent variables: Depression and BMI).

Tables 3, 4 show the regression outputs for BMI and depression. Both tables show that using nutrition apps among women does not significantly impact BMI and depression. Moreover, using fitness apps helped Malaysian women reduce their BMI and depression. However, if we use SEM and include both fitness and nutrition apps in a single model to estimate depression and BMI, we can see different results. Figure 1 shows there is a significant correlation between the usage of fitness apps and nutrition apps, and both fitness and nutrition apps have significant effects on both depression and BMI.

**Table 3:** Regression outputs (dependent variable: BMI).

Variables	Coefficients	Standard error	*t*-Stat	*p*-Value
(Constant)	28.386	1.195	23.761	0.000
F-App	−1.624	0.142	−11.465	0.000
N-App	−0.004	0.146	−0.030	0.976
Screen Time	0.370	0.168	2.200	0.028
Sleeping	0.398	0.153	2.604	0.009
Demographic	0.104	0.215	0.485	0.627

**Table 4:** Regression outputs (dependent variable: Depression).

Variables	Coefficients	Standard error	*t*-Stat	*p*-value
(Constant)	14.411	1.077	13.386	0.000
F-App	−0.550	0.128	−4.307	0.000
N-App	−0.008	0.131	−0.611	0.541
Screen Time	0.313	0.152	2.059	0.039
Sleeping	0.404	0.138	2.927	0.034
Demographic	0.213	0.194	1.101	0.271

### 3.2. Taguchi method analysis

Based on the Taguchi method, data analysis was performed on the primary dataset extracted for the Taguchi experiment. In this study, all the variables were taken into consideration for the Taguchi experimental analysis. However, three indicators, including physical activity and average working hours, were eliminated from the analysis for the lifestyle variable. According to the previous studies, the average screen time used and sleeping hours were retained in the analysis (Khajeheian et al., 2018). For the demographic variable, we calculated the average level of the respondents’ backgrounds and distributed them into five categories, as indicated in Table 2. Each variable has five levels. Hence, the L_25_ (5^5^) Taguchi experimental design was utilized. Table 5 contains the Taguchi coding structure for data analysis using the MINITAB software.

**Table 5:** Taguchi method coding.

Level	Coding	Level	Coding
Demographic (Average)		N-App	
Very Low	Code “1”	Never	Code “1”
Low	Code “2”	Monthly	Code “2”
Moderate	Code “3”	Weekly	Code “3”
High	Code “4”	Every 2–3 days	Code “4”
Very High	Code “5”	Daily	Code “5”
F-App		Screen time	
Never	Code “1”	Less than 1 h	Code “1”
Monthly	Code “2”	1–2 h	Code “2”
Weekly	Code “3”	2–3 h	Code “3”
Every 2–3 days	Code “4”	3–4 h	Code “4”
Daily	Code “5”	More than 4 h	Code “5”
Sleep Amount			
Less than 6 h	Code “1”		
6–7 h	Code “2”		
7–8 h	Code “3”		
8–9 h	Code “4”		
More than 9 h	Code “5”		

**Figure 2:**
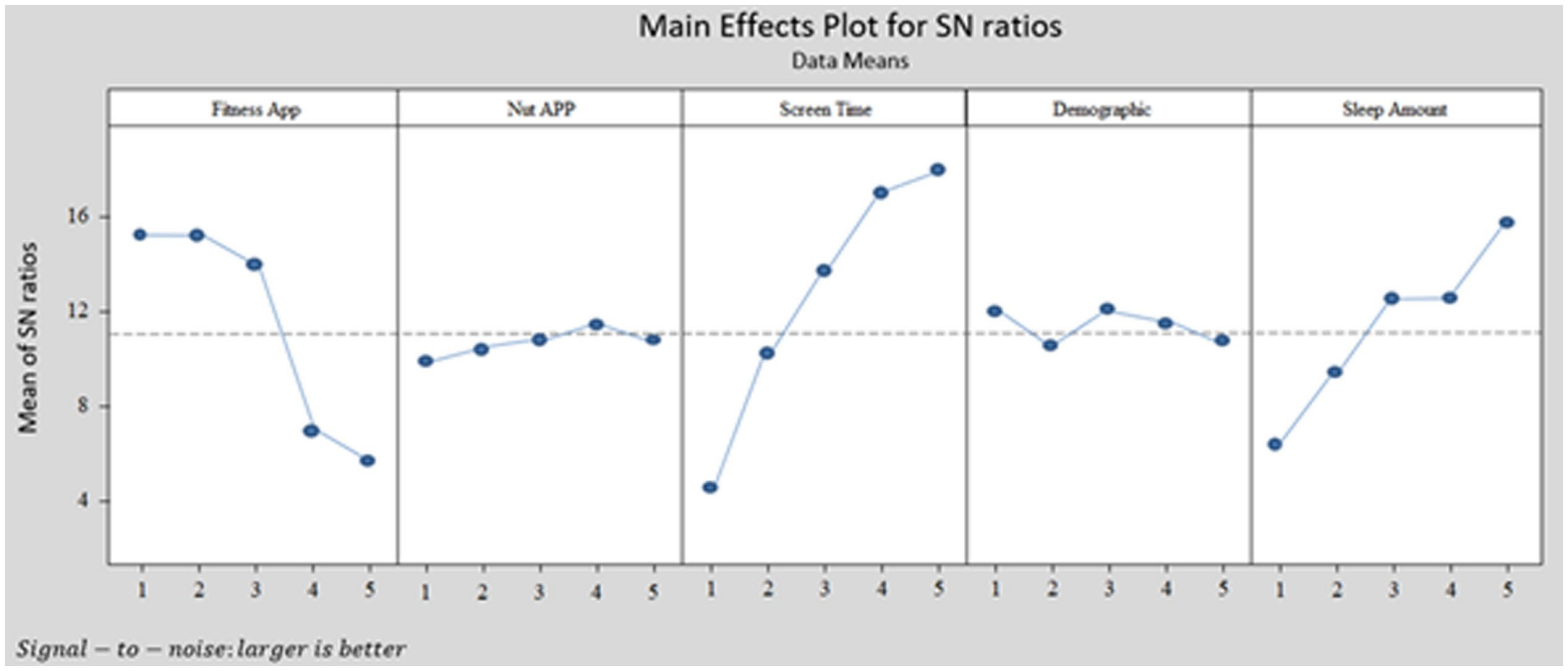
Taguchi output for BMI pattern with MINITAB software.

Figures 1, 2 and Tables 6, 7 express the Taguchi method outputs from MINITAB software for BMI and depression levels, respectively.

**Table 6:** Taguchi output for BMI.

A	B	C	D	E	BMI1	BMI2	BMI3	BMI4	BMI5	BMI6	SNRA	MEAN
1	1	1	1	1	23.2	18.4	30.5	24.2	17.2	22.2	26.653	22.637
1	2	2	2	2	31.2	32.1	20.0	20.8	30.8	23.4	27.915	26.369
1	3	3	3	3	25.9	19.8	26.4	24.0	31.3	29.3	28.045	26.103
1	4	4	4	4	24.5	27.1	27.0	30.6	30.1	17.1	27.788	26.086
1	5	5	5	5	23.6	26.2	24.4	21.6	29.7	28.4	28.019	25.624
2	1	2	3	4	27.4	24.6	20.0	27.1	24.0	19.9	27.319	23.814
2	2	3	4	5	26.5	20.0	21.3	20.3	17.0	30.8	26.624	22.667
2	3	4	5	1	17.8	22.0	32.8	30.1	20.1	26.8	27.321	24.946
2	4	5	1	2	20.2	28.6	19.7	19.1	17.8	32.0	26.608	22.913
2	5	1	2	3	18.3	22.0	29.1	29.4	27.6	25.7	27.692	25.361
3	1	3	5	2	22.2	23.8	22.6	20.1	28.0	17.0	26.654	22.275
3	2	4	1	3	30.7	29.3	24.6	31.4	23.4	32.8	28.939	28.686
3	3	5	2	4	30.6	19.3	26.7	32.6	19.3	28.3	27.775	26.133
3	4	1	3	5	31.4	24.5	24.5	21.2	27.7	31.7	28.309	26.856
3	5	2	4	1	24.4	31.6	32.7	17.8	19.9	27.9	27.528	25.705
4	1	4	2	5	25.4	29.5	26.1	25.0	23.2	23.6	28.038	25.457
4	2	5	3	1	30.9	31.6	31.8	27.9	29.5	23.6	29.17	29.227
4	3	1	4	2	25.9	31.4	23.8	17.1	30.6	27.5	27.755	26.063
4	4	2	5	3	25.5	18.5	17.8	24.8	20.6	32.6	26.8	23.299
4	5	3	1	4	26.8	26.0	19.8	18.1	27.8	31.7	27.462	25.046
5	1	5	4	3	27.1	32.8	26.1	28.0	24.5	24.7	28.571	27.201
5	2	1	5	4	18.4	26.4	21.8	22.1	26.0	29.5	27.302	24.032
5	3	2	1	5	23.6	24.5	21.5	31.8	29.6	33.0	28.405	27.335
5	4	3	2	1	21.4	20.5	17.2	25.9	21.0	24.9	26.537	21.823
5	5	4	3	2	27.5	27.5	21.0	18.6	28.9	31.2	27.764	25.774

**Table 7:** Taguchi output for depression.

A	B	C	D	E	Dep1	Dep2	Dep3	Dep4	Dep5	Dep6	SNRA	MEAN
1	1	1	1	1	26	23	29	6	25	22	22.218	21.833
1	2	2	2	2	6	10	21	34	7	33	19.841	18.5
1	3	3	3	3	35	25	17	23	34	35	28.028	28.167
1	4	4	4	4	18	6	24	34	24	5	19.019	18.5
1	5	5	5	5	21	18	21	29	20	10	24.496	19.833
2	1	2	3	4	12	11	7	8	12	27	20.032	12.833
2	2	3	4	5	10	8	15	18	32	15	21.918	16.333
2	3	4	5	1	11	16	24	16	24	25	24.529	19.333
2	4	5	1	2	21	34	31	15	31	30	27.46	27
2	5	1	2	3	28	12	6	27	21	17	21.437	18.5
3	1	3	5	2	35	15	10	15	24	19	23.941	19.667
3	2	4	1	3	22	19	23	17	34	24	26.714	23.167
3	3	5	2	4	30	15	11	32	25	14	24.457	21.167
3	4	1	3	5	26	13	31	29	25	22	26.546	24.333
3	5	2	4	1	33	33	27	20	35	27	28.807	29.167
4	1	4	2	5	18	12	6	27	6	34	19.468	17.167
4	2	5	3	1	31	16	12	11	25	26	24.12	20.167
4	3	1	4	2	14	27	11	5	10	8	18.731	12.5
4	4	2	5	3	34	27	27	24	15	26	27.263	25.5
4	5	3	1	4	16	25	23	10	13	13	23.123	16.667
5	1	5	4	3	12	11	12	12	11	6	19.643	10.667
5	2	1	5	4	15	30	15	21	11	30	24.428	20.333
5	3	2	1	5	32	23	21	7	33	23	23.256	23.167
5	4	3	2	1	9	28	33	25	30	8	22.613	22.167
5	5	4	3	2	32	8	9	26	31	30	22.652	22.667

Figures 1, 2 show the Taguchi output for women’s obesity and women’s depression, respectively. Figure 1 illustrates that the highest BMI occurred in women who never use the fitness apps or use them once a month, with a 4-h average screen time per day and more than 9 h of sleep. However, according to Figure 2, the highest depression level among women can be observed for respondents who never use fitness apps, spend more than 4 h per day on screen, and have an average sleeping time of fewer than 6 h or more than 9 h per day.

## 4. Discussion

### 4.1. Discussion based on Taguchi method

In this study, we designed the Taguchi method based on demographic details, screen time, sleep amount, F-App, and N-App. We chose “Larger is Better” (See Table 1) in Taguchi’s design. We want to know which combination of research variable levels causes higher BMI and depression levels among women. Figures 1, 2 illustrate the MINITAB software output according to the Taguchi method design for BMI and depression levels, respectively. Based on Figures 1, 2, the demographic and N-App are not significant for both outputs. The patterns shown in these two variables are near the dotted lines. Therefore, it can be interpreted that the demographic and N-App do not significantly affect BMI and depression levels. In other words, for women who have higher BMI and depression levels, their demographics, and N-App do not significantly impact their BMI and depression levels.

Figures 1, 2 show that the F-App has a negative slope, and the diagram has high variation. Therefore, F-App is effective in reducing BMI and depression levels. For better understanding, we grouped this variable (F-App) into groups: group 1 = never; group 2 = monthly; group 3 = weekly; group 4 = 2–3 times per week, and group 5 = more than three times per week. Figure 1 shows that the outputs for groups 1, 2, and 3 are the same in reducing depression. However, there is a significant decrease between groups 3 and 4, and a slight decrease from group 4 to group 5. As a result, if the F-App matches these groups; never, monthly, and weekly, the outputs regarding reducing depression levels are the same, and second, there is no significant effect on reducing depression levels. Females who were using the F-App 2–3 times per week showed better progress in reducing depression. Figure 2 shows similar outputs for groups 1 and 2. However, there is a significant difference between group 2 and group 3 and also between group 3 and group 4. Note that there are no significant differences between groups 4 and 5. As a result, females who use F-App weekly, particularly 2–3 times per week, have a higher likelihood of reducing their BMI over time.

The third diagram (from left) of Figures 1, 2 shows the impact of screen time on depression level and BMI, respectively. The diagram has a positive slope and high variation. We grouped this variable into groups: group 1 = less than 1 h; group 2 = 1–2 h; group 3 = 2–3 h; group 4 = 3–4 h; and group 5 = more than 4 h per day. The diagrams of screen time from Figures 1, 2 show that BMI and depression levels increase drastically with increasing screen time. However, respondents who spend less than 2 h (group 1 and group 2) on screen time have low depression symptoms. If they spend more time on-screen, the findings indicate that they have a higher depression level. This result is supported by Kim et al. (2017)’s previous studies in which smartphone addiction positively correlates with depression. The findings of this study suggest that more screen time increases respondents’ levels of BMI. Respondents who sleep more per night would have a higher BMI as well. This output is supported by previous studies (Maddahi et al., 2020; Salarzadeh Jenatabadi et al., 2020).

The last diagrams of Figures 1, 2 show how sleep amount matters in measuring women’s BMI and depression, respectively. When the women have an average of 7–8 h or 8–9 h of sleep, it will not change their BMI levels. To have a normal BMI range, sleeping between 7 and 9 h is not effective at all. The Taguchi output for depression levels is different compared to the BMI level output. Respondents who use fitness apps every 2–3 days or daily seem to have lower depression levels. The use of fitness apps regularly decreased the level of depression among women. The Taguchi output in Figure 2 for sleeping hours shows exciting patterns. Respondents who sleep less than 6 h or more than 9 h every day have the highest level of depression (i.e., likely depression). The impact of these two levels of sleep on depression levels is the same. Sleep quality might be one of the main possibilities for respondents to suffer from depression. Besides, previous studies also claim that depression is linked to women’s lifestyle choices, e.g., sleep quality (Yang et al., 2019). Berk et al. (2013) abridged that claim by stressing that poor lifestyle choices cause depression.

### 4.2. Discussion based on SEM

We find a strong and consistent link between obesity and depression in this sample of Malaysian women. Additionally, we noticed a stepwise increase in both directions: increasing body mass index was strongly associated with higher risk of depressive disorder and increasing severity of depressive symptoms was strongly associated with higher risk of obesity. We discover that the link between depression and obesity was not just present in cases of more severe obesity. Accounting for potential confounders like demographic, screen time, sleep time, and use of mobile applications for nutrition and fitness had a negligible impact on this association. The correlation could not be explained by the specific effects of obesity on somatic depression symptoms. The demographic groups where the obesity-depression association is strongest may have been the focus of our survey. Despite the fact that we did not find differences in the relationship between obesity and depression across demographic groups, earlier studies have suggested that this connection may differ based on ethnicity, education, age, and monthly income (Gavin et al., 2010; Assari et al., 2014; Lincoln, 2019).

We investigate two likely mediators of the link between obesity and depression: screen time and sleep time. In our study, after controlling for the link between sleep duration and obesity, depression was found to be independently associated with less sleep. Of course, both hypotheses—that obesity causes depression or that depression causes obesity—are consistent with this observation. In the first scenario, depression might cause less sleep, which would then result in weight gain. In the latter case, fewer hours of sleep brought on by obesity may be a factor in low mood. In either case, though, our data imply that the amount of sleep that a person gets may play a role in mediating this relationship. After taking into account the connection between screen time and BMI, screen time was also linked to depression. This result supports the idea that obesity causes depression by stigmatizing the condition and reducing screen time.

### 4.3. Comparison of Taguchi outputs with ANOVA, regression analysis, and SEM process

Can fitness and nutrition apps help women control their BMI and depression during the COVID-19 breakdown? From Tables 3–5 and Figures 1, 2, we can deduce that using nutrition apps does not help women to control BMI and depression levels during the COVID-19 outbreak. However, using fitness apps can increase the frequency of usage and may lower BMI and depression levels among women. From Tables 4, 5, the ANOVA and regression methods show us which variables effectively identify BMI and depression levels. It is worth noting that the Taguchi method gave information that ANOVA and regression could not present. Significantly, the Taguchi outputs of Figures 1, 2 show which combinations of research variables based on their level effectively identify women with higher BMI and depression. However, Figure 1 shows that, with considering both F-APP and N-App for estimating both depression and BMI in a same model, we will have different outputs compare Taguchi method, regression, and ANOVA.

### 4.4. Contributions of the study

**Figure 3:**
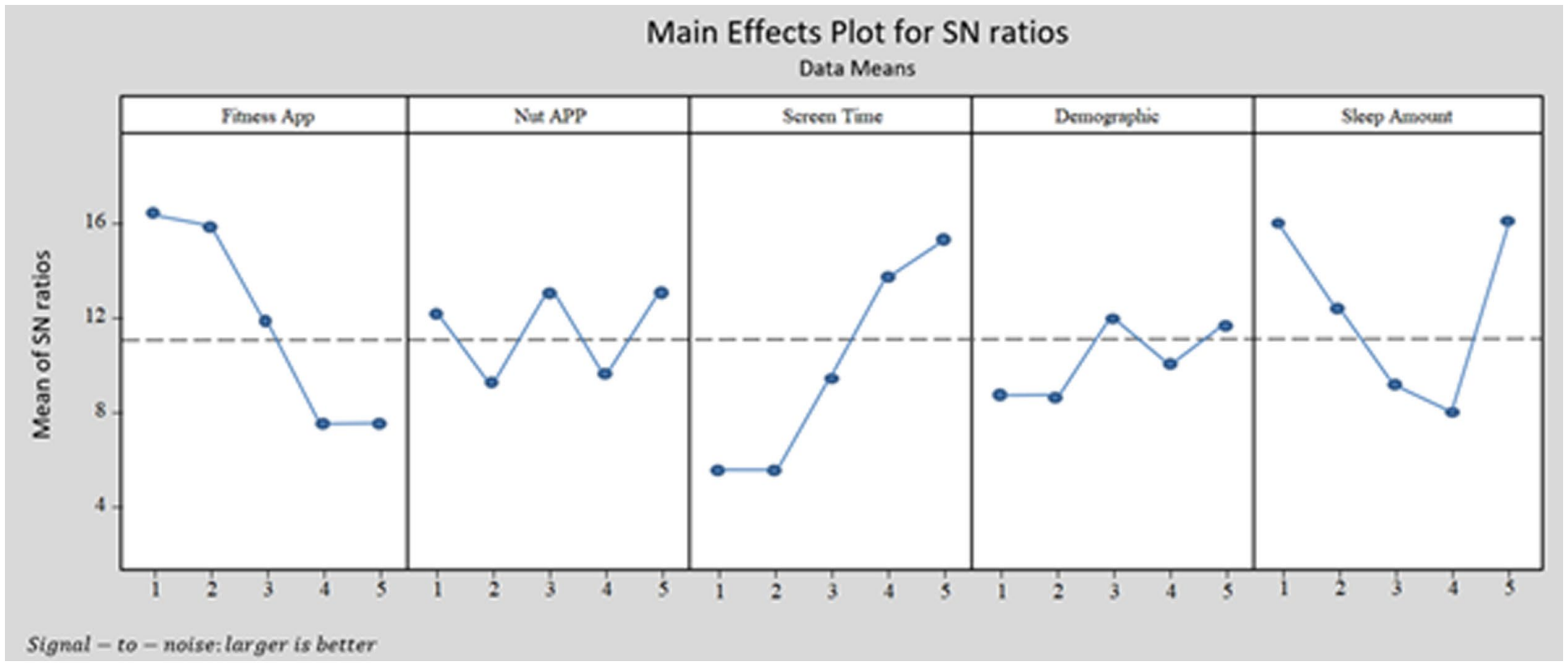
Taguchi output for depression pattern with MINITAB software.

The novel contribution of this paper to theory are multifield. First, and the main contribution, the study adds its valuable contribution the effectiveness of different ways of estimating correlation among research variables with depression and BMI. With considering correlation or effectiveness of F-App and N-App on BMI and depression, ANOVA, regression, and Taguchi method were useful. However, these methods are not able to evaluate both F-App and N-App with other research variables on both BMI and depression in a single model. As you can see in data analysis part for ANOVA analysis we have to analyze the effectiveness of F-App and N-App separately and for every of dependent variables need to present different analysis (see Table 2). This weakness of the analysis can be seen in regression (see Tables 3, 4) and Taguchi (see Figures 2, 3). Therefore, for analyzing one dependent variable analysis, these methods might be applicable. However, estimating two or more than dependent variables in a single model or analysis is applicable by involving SEM.

Second, this study adds valuables results to the mHealth literature. The study revealed demographic does not have significant impact on both depression and BMI based on regression, Taguchi method, and even SEM. However, with SEM technique, we realized demographic has an indirect significant effect on depression through screen time. The sudden and unexpected changes way of analysis, we are able to find hidden significant relationships whish have not been considered before.

Third, another novel contribution of the study is that we found out there is a strong correlation between the usage of F-App and N-App based on SEM technique which has nobody have done before.

### 4.5. Study restrictions

#### 4.5.1. This research also has a few limitations

Self-reported: The questionnaires were self-reported by respondents, especially the weight and height measurements, to determine their BMI. The CES-D and frequency of using mHealth were also self-reported. The validity of this method has been verified in previous studies (Sanchez et al., 2010; Banting et al., 2014). So, we considered the research data acceptable to be analyzed.

The CES-D questionnaire: It should be noted that in this study, we only analyzed the depression symptoms of the respondents. As we calculated the score, we grouped the respondents according to their depression levels, as described in the method section. The questionnaire is not a substitute for clinical diagnosis.

Additional treatments: Some of the respondents might also receive other additional treatments such as personal trainers or psychologists, which could probably contribute to the BMI and depression levels. Future research should look into how these indicators affect female obesity and depression.

## 5. Conclusion

We conclude that throughout the pandemic in Malaysia, using fitness apps consistently was more effective than using nutrition apps to establish a better quality of life among women. As we live in the era of ICT, the availability of thousands of mHealth apps would help people organize their daily lives. For example, fitness apps help promote suitable physical activity, diet control, weight management, stress relief, and sleep monitoring (Higgins, 2016). Apart from that, we believe that poor sleep is also correlated with women’s depression and obesity development, which has been proven in previous studies (Saucedo et al., 2021) and extensive screen time usage. The Taguchi method, which we used in this study, gave public health researchers a way to look at the levels of obesity and depression in women. The last and the main conclusion of this studies, with application of SEM, research scholar are able to find out significant different correlations among research variables compare to familiar mythologies include ANOVA, regression, and Taguchi methods.

## Data availability statement

The raw data supporting the conclusions of this article will be made available by the authors, without undue reservation.

## Ethics statement

The studies involving human participants were reviewed and approved by University of Malaya Research Ethics Committee (UMREC). The patients/participants provided their written informed consent to participate in this study.

## Author contributions

NM, AA, and HJ contributed to conception and design of the study. NM, AA, and HJ organized the database and performed the statistical analysis. NS, NA, and SA wrote the first draft of the manuscript. NM, AA, NA, SA, NS, and HJ wrote sections of the manuscript. All authors contributed to manuscript revision, read, and approved the submitted version.

## Funding

This research is funded by Universiti Malaya Research Grant (Grant No. GPF083B-2020) and Prince Sultan University.

## Conflict of interest

The authors declare that the research was conducted in the absence of any commercial or financial relationships that could be construed as a potential conflict of interest.

## Publisher’s note

All claims expressed in this article are solely those of the authors and do not necessarily represent those of their affiliated organizations, or those of the publisher, the editors and the reviewers. Any product that may be evaluated in this article, or claim that may be made by its manufacturer, is not guaranteed or endorsed by the publisher.
